# Immobilisation of living coral embryos and larvae

**DOI:** 10.1038/s41598-019-51072-5

**Published:** 2019-10-10

**Authors:** Carly J. Randall, Christine Giuliano, David Mead, Andrew J. Heyward, Andrew P. Negri

**Affiliations:** 1 Australian Institute of Marine Science, PMB 3, Townsville, Queensland 4810 Australia; 2 Australian Institute of Marine Science, Indian Ocean Marine Research Centre, University of Western Australia, 39 Fairway Street, Crawley, 6009 WA Australia

**Keywords:** Animal physiology, Embryogenesis, Developmental biology

## Abstract

Embedding and immobilisation of living cells and microorganisms is used in a variety of research and commercial applications. Here we report the successful extended immobilisation of coral larvae in a low-gelling temperature agarose. Embryos and larvae of five broadcast-spawning Scleractinian species were immobilised in agarose gel and tested in a series of exploratory survival and settlement assays. The optimal developmental stage for immobilisation was after ciliation at approximately 24 hours post-fertilisation, after which, survival of immobilised larvae of all species was nearly 100%. In long-term assays, 50% of *Montipora digitata* larvae survived immobilised for 89 days. Furthermore, immobilised larvae of multiple species, that were released from the agarose, generally remained capable of settlement. These results demonstrate that the immobilisation of the early life-history stages of corals is possible for a variety of applications in basic and applied science.

## Introduction

Immobilisation is a well-established biotechnology with the potential to be harnessed for coral-reef research and restoration. Immobilisation is the process of entrapping and restricting the movement of living cells or organisms in a medium, often a hydrogel^1^. Hydrogels are ideal for immobilisation for several reasons: they retain water without dissolving, solute transport through the gel can be regulated by pore size, they resist degradation, and they have anti-fouling properties^2^. The immobilisation of a wide variety of organisms has been used for decades in the pharmaceutical, agricultural, food, and biomedical research industries. For example: bacteria immobilised in a hydrogel have been used in anti-fouling agents^2^; marine diatoms and microalgae have been encapsulated in alginate beads for aquaculture food-delivery systems^3^; *Aiptasia* sp. were immobilised in hydrogel for *in vitro* fertilisation and microinjection^4^; filamentous fungi were microencapsulated for large-particle flow cytometry^5^; and nematodes and squid hatchlings were immobilised in a hydrogel for extended microscopy^6^.

Immobilisation can provide improved control over and survival of cells and living organisms, and when combined with innovations in biomaterials for microencapsulation, can enable delivery systems^5^. Advances in coral breeding along with the predicted advantages of using sexually produced corals for restoration have stimulated research into methods for the collection and controlled delivery of coral larvae onto degraded reefs^7–9^. However, larval and post-settlement survival rates remain low after deployment, and current techniques are not well suited for the co-deployment of larvae with any form of enrichment, such as probiotics, nutrients, or endosymbiotic dinoflagellates. Immobilisation and microencapsulation have the potential to be applied to such delivery systems. Therefore, the objectives of this study were to (1) evaluate the effect of immobilisation on coral embryo and larval survival, and (2) test post-embedding settlement success.

## Results and Discussion

The immobilisation of coral embryos and larvae was tested across a range of concentrations of low-gelling temperature agarose. Concentrations of 1.0–1.25% provided the best structural integrity of the gel but set at higher temperatures (~31–32 °C), whereas concentrations of 0.65–0.8% resulted in less structural integrity and poorer embedding but set at cooler temperatures (~28–29 °C). Successful embedding rendered larvae completely immobile (i.e. ‘suspended’ in gel and unable to swim). While 31 °C is warmer than normal summer temperatures on the Great Barrier Reef, a short-term exposure to 31 °C had no observable negative impacts, and temperatures cooled to 28 °C within ten minutes of encapsulation. Hardening of coral offspring by exposing them to heat stress has been proposed as a technique to increase thermal tolerance of corals^10^; controlled larval immobilisation at higher temperatures, therefore, may serve a dual purpose by stress-hardening during the encapsulation process. Yet, whether this transient exposure to heat stress can result in lasting effects must be tested experimentally.

**Figure 1:**
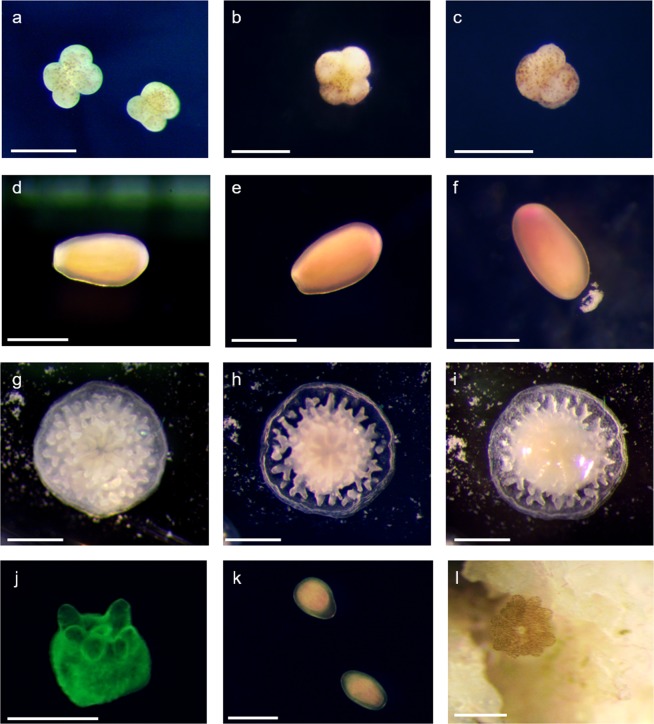
*Montipora digitata* 4-cell embryos immediately (**a**), 24 hours after (**b**), and 7 days after (**c**) embedding. *Acropora millepora* larvae immediately (**d**), 24 hours after (**e**), and 4 days after (**f**) embedding. *Acropora millepora* spat immediately after (**g**), 4 days after (**h**), and 7 days after (**i**) embedding. (**j**) *Montipora digitata* metamorphosed in gel matrix and imaged using fluorescence microscopy. (**k**) *Platygyra daedalea* in gel matrix immediately after embedding. (**l**) *Montipora digitata* settled on a rubble fragment after 89 days immobilised. Scale bars = 0.5 mm.

**Figure 2:**
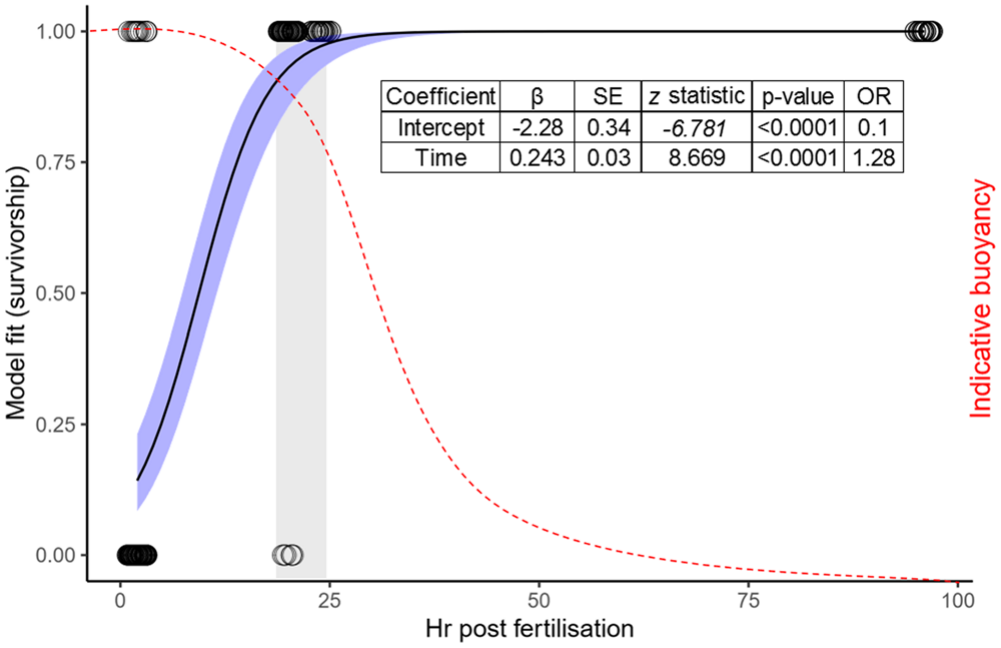
Survival of agarose-embedded *Montipora digitata* embryos (points are jittered horizontally for clarity) plotted against hours after fertilisation with a logistic model fitted to the data (black curve). Blue shading represents the 95% confidence interval around the model. Red curve indicates the indicative buoyancy of embryos/larvae for many coral species. Grey shading indicates optimal theoretical window for embedding from 19–24 hr corresponding with an estimated 95–99% survival and high larval buoyancy. Model coefficients are presented in the inset table. SE = standard error; OR = odds ratio.

**Figure 3:**
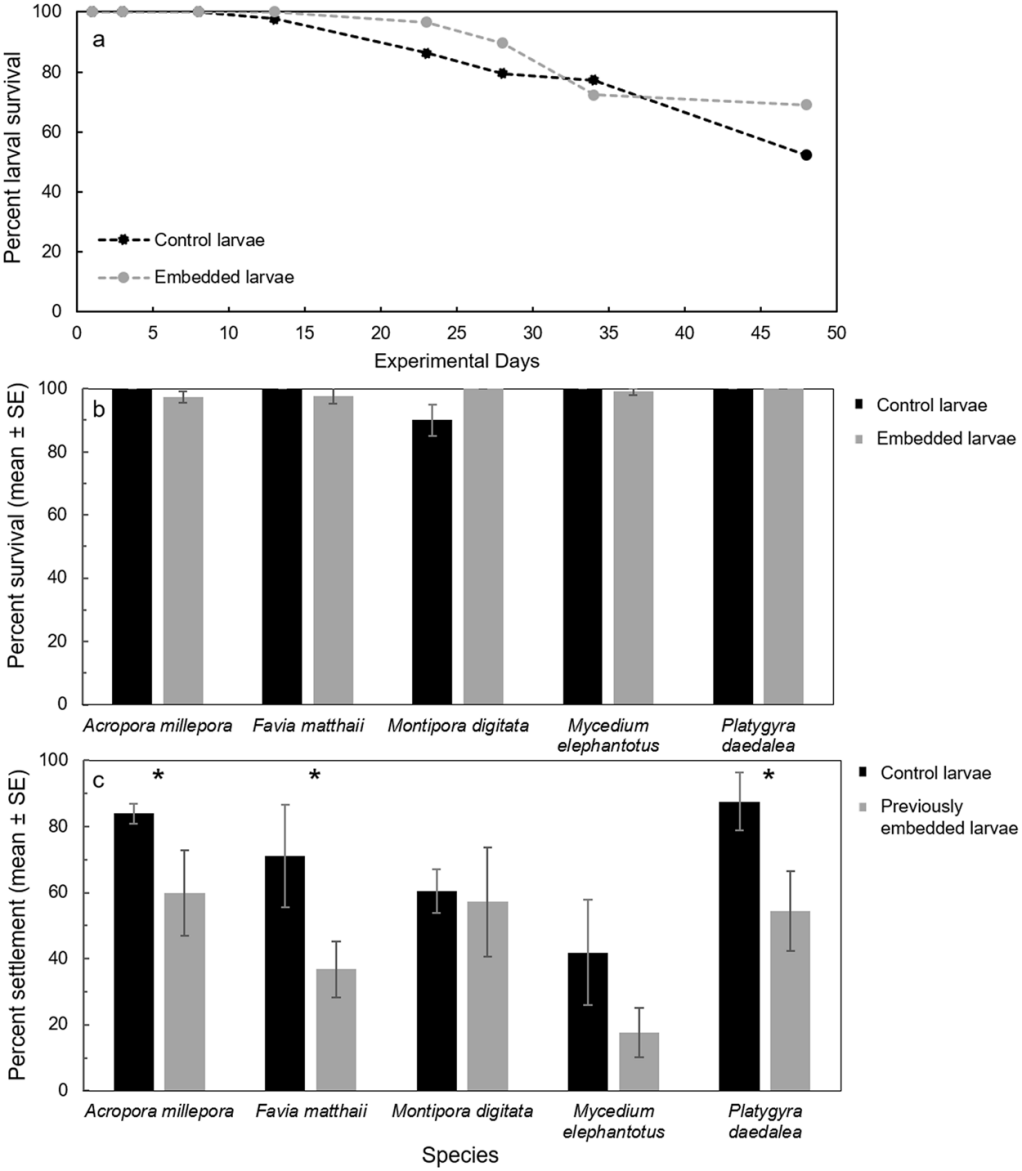
(**a**) Long-term survival of agarose-embedded and control *Montipora digitata* larvae in individual wells of 48-well plates (n = 48 larvae per treatment). (**b**) Average percent survival of agarose-embedded and control larvae tested in 3– to 6–day assays. (**c**) Average percent settlement of previously embedded and control larvae tested in 24–48 hr assays. * indicates statistically significant differences (p ≤ 0.05) between treatments for a given species.

Immobilisation during very early development appeared to halt embryogenesis. Immobilised 4-cell embryos remained in an apparent 4-cell stage, indicating either a cessation of cell divisions, or a continuation of cell divisions in an embryo that was shaped as a 4-cell morula (Fig. 1a–c). After several days, immobilised early embryos began to disintegrate, and survivorship was poor (Fig. 2). Conversely, when early-stage larvae were immobilised after they had developed cilia (Fig. 1d–f), survival rates increased to nearly 100% and were comparable with controls (Figs 2, 3b). In a long-term assay, survival rates remained near 100% for over two weeks before slow attrition began in all treatments, including seawater controls (Fig. 3a). Even after 89 days embedded, ~50% of *Montipora digitata* larvae in agarose were alive. Immobilised larvae appeared to survive by creating a mucous cocoon, which allowed ciliary action to continue, presumably enabling the exchange of gases and the movement of particulate organic material (Fig. 1f, Supplementary Video). This movement, which allowed the larvae to rotate in place, was observed during encapsulation, as was reported by Jones *et al*.^4^ for *Aiptasia* sp. larvae, and suggests that immobilised ciliated larvae can modify their microenvironments (Supplementary Video). This protective mechanism is like that described for *Acropora* spp. embryos, which form protective cocoons in response to suspended sediments^11^. We suggest that the optimal timing of coral encapsulation is, therefore, after embryos are ciliated, but prior to decline in buoyancy, for ease of collection (Fig. 2). These results indicate that immobilisation could serve as a research tool for studies of both coral embryos and larvae; embedded embryos remain alive for several days and larvae for weeks to months, with each organism individually trackable, imageable, and easily manipulated. As such, gel immobilisation offers an alternative to standard ‘fixation’ methods.

*M*. *digitata* provides photosynthetic endosymbionts to eggs prior to spawning, whereas the larvae and juveniles of the other species tested take up symbionts from the environment. No differences in embryo or larval survival were observed over 3–6 days among the species tested, suggesting that symbiotic and aposymbiotic larvae respond similarly to immobilisation. We hypothesize that the presence of endosymbionts contributed to the longevity of *M*. *digitata* in the long-term experiment, by providing oxygen and other photosynthates to the larvae for nutrition^12^. While co-embedding with symbiotic dinoflagellates was not tested in this study, there is precedence for the encapsulation of similarly sized, single-celled marine diatoms and microalgae^13,14^, suggesting that encapsulation of Symbiodiniaceae is possible. Co-encapsulation with a facilitator may enhance survival or performance of coral larvae^15,16^. The co-encapsulation of yeast with microalgae, for example, increased yeast biomass and lipid productivity by 1.6-fold over mono-encapsulation^15^. Future coral co-encapsulation techniques could be used to (i) inoculate larvae with stress-tolerant Symbiodiniaceae, (ii) provide probiotics that increase larval and recruit fitness, and (iii) incorporate food for heterotrophic larvae to increase survival and post-settlement growth.

While immobilisation in hydrogel was successful, agarose does not dissolve in seawater. Therefore, to evaluate post-embedding settlement, larvae were manually removed from the gel. Most larvae elongated and swam off within a few seconds of being freed from the gel. The swimming behaviour and morphology of released larvae appeared normal. If any gel remained in contact with a larva after extraction, however, it remained immobile. No significant differences in settlement between previously embedded and control larvae were observed for *Montipora digitata* or *Mycedium elephantotus*, although settlement rates on average declined by 35% compared with controls across all species, and were significantly reduced for *Acropora millepora*, *Favia matthaii*, and *Platygyra daedalea* (Fig. 3c). We note, however, that any larvae with remnant gel attached were unable to swim, which contributed to settlement failure. Of those larvae that did settle, morphology appeared normal. While not formally tested, larvae were observed settling after 89 days immobilised (Fig. 1l). The development of a mechanism for the controlled and gel-free release of larvae after embedding would clearly improve settlement.

Immobilised *A*. *millepora* spat demonstrated a ‘polyp bail out’-like behaviour (*sensu* Sammarco^17^), whereby tissue slowly retracted from the skeleton and amassed around the mouth over a 7-day period (Fig. 2g–i). Spat also appeared to form a protective mucous coating, yet whether they could successfully ‘bail-out’ and return to the larval stage after extraction was not tested.

In conclusion, immobilisation of coral embryos and larvae in agarose gel was tested across a range of developmental stages, for five Indo-Pacific coral species. Survival rates of embedded larvae were high and comparable with controls when immobilisation occurred after ciliation, and larvae were capable of settling post immobilisation. While the technology to embed and encapsulate coral embryos/larvae for restoration applications requires significant advancement and feasibility testing, this study suggests that coral larvae may be excellent candidates. This manipulation, to isolate and maintain planulae, is amenable to use in almost any laboratory. The immobilisation of larvae in discrete, partitionable micro-chambers may also prompt innovative experimental techniques to investigate variability in larval biology and response to the environment.

## Materials and Methods

### Coral spawning and larval rearing

Gravid corals were collected from Backnumbers Reef (*Acropora millepora*, *Platygyra daedalea*, *Favia matthaii*, *Mycedium elephantotus*) and Orpheus Island (*Montipora digitata*) on the central Great Barrier Reef, approximately one week prior to predicted spawning dates in 2017 and 2018, and were transported to the National Sea Simulator at the Australian Institute of Marine Science. The timing of spawning and the numbers of colonies that contributed spawn to mass cultures are reported in Table 1. Gamete bundles were collected, separated, washed, and fertilized as described in Pollock *et al*.^18^, and mass cultures were maintained in flow-through aquaria at ambient temperature (26–27 °C), with no aeration during the first 24 hours, and low aeration thereafter.

**Table 1:** Summary of spawning corals used to generate mass cultures of coral embryos and larvae tested in immobilisation trials.

Species	Spawn date	no. nights after full-moon	Spawning commencement time(hh:mm)	Total no. spawning colonies
*Acropora millepora*	9^th^ Nov 2017	5	21:15	10
	28^th^ Oct 2018	3	20:40	7
*Favia matthaii*	28^th^ Nov 2018	5	21:20	3
*Montipora digitata*	4^th^ Apr 2018	4	19:21	9
	6^th^ Apr 2018	6	19:24	18
*Mycedium elephantotus*	28^th^ Nov 2018	5	21:06	4
*Platygyra daedalea*	29^th^ Oct 2018	4	18:45	13

### Immobilisation trials

Immobilisation of embryos and larvae was tested in low-gelling-temperature agarose hydrogel (Sigma-Aldrich A9414) at concentrations of 0.65% to 1.25% weight/volume in 0.2 µm filtered sea water (FSW) at 35.1 ppt (mean ± SD 0.6). A range of agarose concentrations was tested to identify the corresponding gelling temperatures in seawater, and to determine the optimal concentration for immobilisation. Embryos/larvae were either pipetted with 50 µl of FSW directly into liquid agarose and gently mixed using the pipette tip (i.e. ‘agar first’ in Table 2) or pipetted into an empty well plate with 50 µl of FSW after which agarose was pipetted on top of the sample (i.e. ‘embryo first’ in Table 2). We note that the 50 µl FSW added with the embryos/larvae was considered an insignificant volume and thus was not accounted for in the reported concentration of agarose. Immobilisation was trialled in 6-well plates, 48-well plates, and petri dishes. The temperature of the agarose was monitored during the trials and gelling temperatures were recorded.

**Table 2:** Summary of conditions and sample sizes for each assay reported.

Assay	Species	Culture date	Assay type	Embryo/larval age	Conc. agarose (%)	Vol. agarose (ml)	Vol. FSW over agarose (ml)	Embryo/larval embedding technique	No. Reps	No. embryos/larvae per replicate (mean ± SD)	Embedded duration (days)	No. days read post settlement induction
Embryogenesis	*Montipora digitata*	6/04/2018	6-well plate	2 hr	0.65	5.0	5.0	embryo first	6	10 ± 0	3	NA
	*Montipora digitata*	4/04/2108	Petri dish	2 hr	1.00	30.0	20.0	embryo first	1	30	2	NA
	*Montipora digitata*	4/04/2018	6-well plate	20 hr	0.80	3.0	7.0	embryo first	9	10 ± 2	4	NA
	*Montipora digitata*	4/04/2018	6-well plate	24 hr	0.80	3.0	7.0	embryo first	6	5 ± 0	4	NA
	*Montipora digitata*	4/04/2018 & 6/04/2018	48-well plate	96 hr & 144 hr	0.80	0.8	0.8	embryo first	29	1	3	NA
Short-term survival	*Montipora digitata*	4/04/2018	6-well plate	1 day	0.65	3.0	7.0	embryos first	6	5 ± 0	4	NA
	*Acropora millepora*	28/10/2018	6-well plate	13 days	0.8	7.0	0.0	agar first	6	11 ± 2	4	NA
	*Favia matthaii*	28/11/2018	6-well plate	36 days	0.8	7.0	0.0	agar first	6	15 ± 1	5	NA
	*Mycedium elephantotus*	28/11/2018	6-well plate	36 days	0.8	7.0	0.0	agar first	6	17 ± 2	6	NA
	*Platygyra daedalea*	29/10/2018	6-well plate	12 days	0.8	7.0	0.0	agar first	6	9 ± 4	4	NA
Long-term survival	*Montipora digitata*	4/04/2018 & 6/04/2018	48-well plate	96 hr & 144 hr	0.8	0.8	0.8	embryo first	29	1	cont.	NA
Settlement	*Montipora digitata*	4/04/2018	6-well plate	17 days	0.8	7.0	0.0	agar first	6	8 ± 1	1	2
	*Acropora millepora*	28/10/2018	6-well plate	17 days	0.8	7.0	0.0	agar first	6	10 ± 1	4	1
	*Favia matthaii*	28/11/2018	6-well plate	41 days	0.8	7.0	0.0	agar first	6	11 ± 2	5	2
	*Mycedium elephantotus*	28/11/2018	6-well plate	42 days	0.8	7.0	0.0	agar first	6	15 ± 1	6	2
	*Platygyra daedalea*	29/10/2018	6-well plate	16 days	0.8	7.0	0.0	agar first	6	10 ± 1	4	1
Spat	*Acropora millepora*	9/11/2017	Petri dish	14 days post settlement	0.7	5.0	5.0	spat first	1	5	7	NA

We note that the volume of agarose (ml) equalled the volume of the filtered seawater (FSW) equivalent in negative controls.

### Larval development

To evaluate the optimal developmental stage for immobilisation, *Montipora digitata* embryos were embedded at the 4-cell stage (2 hr post fertilisation), late gastrula stage (20 hr post fertilisation), early larval stage (24 hr post fertilization) and advanced larval stage (≥96 hr post fertilisation). Survival was modelled as a function of hours post fertilisation using a logistic regression with a binomial distribution and a logit link function using ‘glm’ from the ‘stats’ package in R^19^.

### Larval survival

Larval survival during immobilisation was tested using two sets of assays. Firstly, a short-term (3–6 day) survival assay was completed for all five species. Approximately 10 larvae per well were immobilised in gel in sterile 6-well plates (assay conditions reported in Table 2). Initial numbers of larvae per well were recorded, and survival was assessed after 3–6 days. Larvae were considered ‘alive’ if they (i) were normally pigmented compared with control larvae, (ii) were observed beating their cilia and/or rotating, and (iii) had an intact ectoderm. Secondly, a long-term assay was completed with *M*. *digitata* where a single larva (4–6 days old) was immobilised in wells of a 48-well plate (Table 2). In some assays, including long-term survival, FSW was added on top of the gel to prevent desiccation (Table 2). Control wells were prepared with larvae in an equivalent volume of FSW. Well plates were maintained under AquaIllumination® Hydra light emitting diode (LED) aquarium lights (171 photosynthetically active radiation (PAR), 12:12 h diel cycle) in a temperature-controlled experimental room (27 °C), and the status (i.e. live, dead, metamorphosed) of each larva was evaluated over 48 days; a final reading of only embedded larvae was made after 89 days.

### Larval settlement

To evaluate the effect of previous immobilisation on larval settlement, larvae were embedded in agarose in sterile 6-well plates (Table 2). Control plates were prepared with larvae of the same age in equivalent volumes of FSW. After a period of immobilisation that ranged from 1 to 6 days depending on the species (see Table 2), larvae in each treatment well were carefully released from the gel using dissection tools under a standard dissection microscope, and gently transferred via pipette into sterile 6-well plates with 10 mL FSW. Control larvae were also pipetted into 10 ml FSW into new sterile plates. All wells were then provided with a fragment of ‘live’ reef rubble (~1 cm^2^; all fragments in a given assay were from the same parent fragment) to initiate settlement. Percent settlement in each well was assessed after 24 hr and percent settlement of previously immobilised larvae was compared with control larvae using a Welch’s two sample t-test in R (R Core Team 2019). We note that all survival and settlement assays were carried out with only embryos/larvae that were, or had been, fully immobilised.

### Spat

Lastly, five *A*. *millepora* recruits (<7 days post settlement) attached to clean glass tiles^20^ were embedded in agarose and observed over seven days. Tiles with living spat were placed in a sterile petri dish. Five millilitres of 0.7% weight/volume agarose in FSW was pipetted into the petri dish at approximately 30 °C. The agarose quickly cooled and gelled, after which five millilitres of FSW was pipetted to cover the layer of gel. The petri dish was maintained in a temperature-controlled experimental room (27 °C), as described above, and assessed for viability each day.

### Compliance with ethical standards

All applicable international, national, and/or institutional guidelines for the care and use of animals were followed. Corals were collected under permit # G12/35236.1 issued by the Great Barrier Reef Marine Park Authority.

## Supplementary information

Supplementary video

## Data Availability

All data are available upon request.
